# The social value of place‐based creative wellbeing: A rapid review and evidence synthesis

**DOI:** 10.1111/1467-9566.13827

**Published:** 2024-08-17

**Authors:** Rafaela Neiva Ganga, Laura Davies, Kerry Wilson, Margherita Musella

**Affiliations:** ^1^ Liverpool Business School Liverpool John Moores University Liverpool UK; ^2^ School of Humanities and Social Science Liverpool John Moores University Liverpool UK; ^3^ Social Research Institute University College London London UK

**Keywords:** arts & wellbeing, creative health, cultural mega‐events, place‐based art interventions, social capital, social inequalities, social value

## Abstract

Creative well‐being is an increasing field of interest to which biomedical and social sciences have made uneven contributions. The instrumental value of culture and its subsequential public investment is grounded in the interplay of social, cultural and economic capital to attain and preserve wellbeing and health and foster social mobility. The current evidence addresses the effectiveness of arts interventions in improving illnesses. Little attention has been paid to the social value of creative wellbeing for the general population. This paper is a rapid review and evidence synthesis that aims to answer the question, ‘What is the social value of place‐based arts and culture interventions at individual (wellbeing) and community (social inequalities) levels in the UK and Europe?’. After a systematic search of five databases, search engines, and a call for evidence in August 2022, 14 out of 974 sources met the inclusion criteria. Studies were organised into three themes (*Community*, *Events*, *Museums*), and outcomes were analysed considering the indicators and dimensions of wellbeing (Office for National Statistics). The review evidenced that creative wellbeing leads to improvements in wellbeing outcomes and can contribute to alleviating social determinants of health. However, considering their impact on the underlying causes of structural social inequalities requires caution.

## INTRODUCTION

Culture, health and wellbeing is an increasingly active field of interest that combines nearly 30 years of research from the health and social sciences. Studies on creative health have mainly focussed on the effectiveness of arts interventions in improving illnesses among older adults. Research has reported cautious but positive outcomes on physical and mental health, highlighting critical implications for policy and practice (Bungay et al., 2014; Leckey, 2011; McLean et al., 2011; Wynn Owen et al., 2013). At a global level, there is now substantial evidence based on the role of the arts in preventing ill health and promoting health across the lifespan (Fancourt et al., 2020; Fancourt & Finn, 2019).

Increasingly, cultural institutions and national statistical agencies have begun routinely monitoring engagement with the arts to understand participation trends and their impacts on health and wellbeing (Tymoszuk et al., 2021). In the United Kingdom (UK), the Office for National Statistics (ONS) tracks participation and satisfaction with leisure through its national Measuring Wellbeing Programme, which captures progress in 10 quality of life domains, including ‘our health’, ‘where we live’ and ‘what we do’. More recently, the UK government has brought evidence of the value of arts and culture to health and wellbeing into decision‐making, and health inequalities are now a key consideration in cultural policy‐making.

In the broader arts and health literature, sociological perspectives have been overlooked, favouring biomedical perspectives (Daykin et al., 2017). Existing research primarily concentrates on the effectiveness of arts interventions in improving illnesses, particularly among older adults, neglecting the broader social value of creative wellbeing for healthy populations. Over the past 10 years, an accumulation of reviews of research and evaluation on creative health have been published, contributing to developing an evidence base that aims to inform policy and practice (Bungay et al., 2014; Leckey, 2011; McLean et al., 2011; Wynn Owen et al., 2013). Cautious but positive outcomes have been reported on the value of arts interventions in treating and managing physical and mental health conditions. There are concerns about the quality of primary research and systematic reviews in the field, with scholars calling for more reliable evidence to inform decision‐making (Clift et al., 2021). In addition, there is a notable knowledge gap on the longer‐term effects of arts interventions and wellbeing frameworks, and studies use measures inconsistently. High‐quality evidence is needed to enhance knowledge exchange between research and policy in arts‐based interventions, particularly regarding long‐term effects on individual wellbeing and community social inequalities (Munn et al., 2018).

Our review addresses a gap in creative health research, focussing on creative wellbeing in healthy populations. It explores the under‐researched area of cultural value in non‐diagnosed conditions, aiming to demonstrate how cultural interventions can improve wellbeing and reduce social inequalities. This review adopts a sociological perspective to examine the social value of place‐based art interventions in improving the wellbeing of healthy populations and reducing social inequalities in the UK and Europe. It addresses the research question: ‘What is the social value of place‐based art interventions at an individual and societal level in the UK and Europe?’.

The review combines insights from creative health, arts‐based research and sociological theory to develop a proposition of how, why and for whom these interventions generate a range of individual and societal wellbeing outcomes. A quality‐of‐life framework is used to identify pathways from arts‐based practices to wellbeing outcomes. The study draws on Bourdieu’s constructs of cultural and social capital (Bourdieu, 1982) to study the effects of social inequalities both in determining access and participation in culture. Specifically, it aims to develop research knowledge to support decision‐making in place‐based cultural policy in the following areas: (i) the role of place; (ii) mechanisms of change; (iii) the value of heritage in improving wellbeing; (iv) the role of inequalities in shaping cultural access, and participation, and (v) the contribution of place‐based arts interventions in mitigating social and health inequalities.

## BACKGROUND

### Social, cultural and economic capital as determinants of health

Since the publication of *Closing the Gap in a Generation* over 10 years ago, the health of people living in more deprived areas in England, UK, has worsened as social and health inequalities have increased (Marmot, 2020). Marmot et al. (2008) found that 70% of health outcomes are determined by the circumstances in which people are born, live, study and work. The social gradient in health is determined by the intersection between social class and a series of health indicators. As such, low socioeconomic status (social, cultural and economic capital) is likely to preclude a healthy lifestyle (Marmot et al., 2008).

Bourdieu's theory, particularly his concept of capital, offers a framework for understanding health inequalities (Bourdieu, 1982, 2018a). Cultural, social and economic capital influence an individual's health outcomes. Those with more capital have better access to healthcare and health‐promoting environments, leading to disparities in health across different social classes. Bourdieu’s theory suggests health inequalities are not just a matter of individual lifestyle choices or genetic predispositions, but also a reflection of deeper social structures and unequal distribution of various forms of capital (Bourdieu, 1983). Social class, therefore, is a significant health and wellbeing determinant (Marmot, 2020; Marmot et al., 2010).

According to Bourdieu (2018a), capital is cumulative in its institutionalised or embodied forms, enabling individuals and groups to be inscribed in social structures. People from the same group or community share aesthetic preferences, cultural practices, health, life choices and opportunities, which become markers of their social class (Bourdieu, 2018a).

Institutionalised (education), objectified (possession of cultural goods) and embodied (values and tastes) cultural capital refers to the collection of symbolic elements that one acquires through being part of a particular social class. Institutionalised and embodied forms of cultural capital are health determinants—health‐related knowledge, for example, influences healthy lifestyles and cultural practices across the life course and increases the cognitive reserve that acts as a protective factor in dementia (Fancourt et al., 2018). Hence cultural participation is associated with positive wellbeing outcomes. Social, cultural and economic capital have a cumulative and interchangeable effect, accruing advantages or disadvantages throughout life (Marmot et al., 2010).

Resource social capital (Fulkerson and Thompson, 2008) reflects the work of Granovetter (1973) and Bourdieu (1982, 2018a), for whom social capital is an individual resource, analysed at the individual level (e.g. relationships). It focuses on group membership (collectively owned capital) and networks (acquaintance and recognition) that might provide access to resources. Resource social capital refers to both actual and potential resources tied to a durable network of institutionalised relationships that individuals accumulate, often characterised by institutionalised recognition and mutual understanding (Bourdieu, 1983). These relationships manifest in various forms, including practical, material and symbolic exchanges (Bourdieu, 1983).

Normative social capital (Fulkerson and Thompson, 2008) builds on Putnam, Coleman and Hanifan’s definition of social capital as a collective good to be assessed at the collective level (e.g., community). Halpern’s (2005) conceptual framework further defines social capital as an adhesive that binds individuals together. It emphasises reciprocity, values, norms, trust and other benefits of bringing people together to generate a collective action and improve the efficiency of the social structure, whereby schools, families and communities work together for mutual benefit, fostering individual and social wellbeing.

The UK ONS and His Majesty's Treasury Green Book Supplementary Guidance define social capital as ‘a term used to describe the extent and nature of our connections with others and the collective attitudes and behaviours between people that support a well‐functioning, close‐knit society’ (Treasury, 2021; Treasury, 2022a). This policy‐based definition of social capital emphasises the collective attitudes and behaviours that contribute to a well‐functioning society, aligning with the normative definition of social capital (Fulkerson and Thompson, 2008). This contrasts with the resource perspective (Bourdieu, 1983; Granovetter, 1973), which views social capital as an individual asset based on personal relationships and networks. The former helps to understand the social value of place‐based creative wellbeing, which, as cultural interventions that aim to improve wellbeing and social inequalities, is assessed by measuring social capital as a proxy of its social value. Hence, improvement in the UK’s National Wellbeing Framework (ONS, 2022) indicators, such as generalised trust, are proxies of how cultural participation generates social value. The latter, considering the capitals’ cumulative and interchangeable effect, reinforces the value of cultural practices to generate social capital and vice‐versa and contributes to understanding the social value of creative wellbeing. Bourdieu’s capital theory has extensively underpinned theoretical and empirical analyses of the social determinants of health (Alegría et al., 2018; Collyer et al., 2015; Doblytė, 2019; Shim, 2010). Hence both definitions are used in this review.

Applying Bourdieu’s theory (Bourdieu, 1982, 1983, 2018a) to health inequalities highlights the role of societal factors in health outcomes and the need for policies addressing these determinants. Furthermore, analysing art interventions ‘in place’ is meaningful in understanding the value of culture to improve individual wellbeing and alleviate social inequalities (Popay et al., 2006). The growing body of evidence in creative health that correlates increased social and cultural capital and good health (Fancourt & Finn, 2019) emerges from the valuation of culture and its ancillary effects, which has been the focus of the cultural value debate for over three decades.

### The cultural value debate

In a seminal analysis of the cultural system, Holden (2006) introduced the idea of 'cultural value' as having three components: intrinsic, instrumental and institutional. Intrinsic value is ‘the set of values that relate to the subjective experience of culture intellectually, emotionally and spiritually’ (Holden, 2006); instrumental value ‘relates to the ancillary effects of culture, where culture is used to achieve a social or economic purpose’ (Holden, 2006); and institutional value ‘relates to the processes and techniques that organisations adopt in how they work to create value for the public’ (Holden, 2006).

Public investment in culture to achieve social and economic outcomes and its subsequent justification have become priority topics for academic research, policy‐making and civil society debate (Scott, 2010). In the UK, the cultural policy field has increasingly focussed on the instrumental value of culture, where its ancillary impacts are assessed as a proxy for its value. However, critics of the instrumentalisation of culture argue that focussing on impact is an ‘inadequate single proxy’. It leads to growing pressures for cultural commodification, reducing its public value and impoverishing the debate. Still, different types of evidence on the value of culture in achieving social (e.g. mental health and wellbeing) or economic purposes have been used to argue for public investment in culture (Belfiore, 2012, 2015; Belfiore & Bennett, 2009; Gilmore, 2014; Holden, 2006).

The belief that culture positively affects health and wellbeing has developed from multidisciplinary perspectives. The positive effect of aesthetic experiences that help individuals to make meaning of their life experiences comes from a philosophical approach. Its effects on brain structure and cognitive functioning originate in health research. Its positive impact on emotional regulation is shown in psychological studies. Finally, sociological research indicates that cultural capital as a social distinction and a symbolic resource can be used to improve health and life opportunities (Pinxten & Lievens, 2014).

Theoretical contributions to cultural value also emerge from economists and sociologists (Crossick and Kasznska, 2016; Hennion, 2005; Lamont, 2012; O'Brien, 2010; Throsby, 2001). Economic approaches focus uniquely on instrumental value, creating a narrative that values culture only for its mercantile effects (Throsby, 2001). Sociology of culture, valuation and other interdisciplinary and applied perspectives contributed to the debate by arguing for the ancillary social effects of culture, shifting the debate, for example, towards intrinsic and organisational value (Campbell et al., 2017; Walmsley, 2018).

However, methodological and epistemological issues emerge as different actors in the cultural field (i.e. producers, or receptors) define and evaluate cultural value in different ways. Heterogenic perceptions and experience of instrumental value from different places in the social field make the integration and standardisation of valuation methods challenging (Boltanski & Thévenot, 2006; Hennion, 2005; Lamont, 2012) Despite recent developments in creative health, establishing a causal link between culture and a beneficial economic or social impact is problematic since interventions do not happen in a vacuum. There are issues already raised by Holden (2006) of ‘temporal remoteness, the complexity of the interaction, the context in which it takes place and the multiplicity of other factors impacting the result’. As such, there is a lack of robustness and replicable evidence of the power of cultural interventions to economically regenerate places and change individuals’ and communities' social conditions (Selwood, 2005). There is a significant distance between arguing for the transformative value of culture and generating the robust evidence required by the Green Book (Treasury, 2022b). Even if cultural interventions work, in the sense of generating ancillary effects, the tensions within instrumental rationality remain. The historical policy‐driven emphasis on the economic value of culture (Belfiore, 2012) has limited the extent to which the instrumental social value of culture (Reidpath et al., 2005), especially its impact on wellbeing, can be measured and advocated.

The economic value of culture has been a driving force in cultural policy‐making, particularly in more conservative approaches to culture‐led economic regeneration (Ganga et al., 2021). The hosting of cultural events (e.g., European Capital of Culture), or capital investment in cultural infrastructure (e.g., a new theatre or gallery), is expected to generate a significant economic return on investment for host cities and urban environments, usually via enhanced tourism and visitor spend. While definitive conclusions on the social value of hosting cultural events are elusive (Garcia & Cox, 2013; Ooi et al., 2014), there is some evidence of increased social capital during hosting, with legacy outcomes being short‐lived (Garcia et al., 2010), or linked to specific programmes, such as community projects and volunteering (Culture, 2018b; Liu, 2014, 2017; Tjarve & Zemīte, 2016). Lengthy theoretical and empirical debate has been dedicated to demonstrating the social risks of economically driven cultural policy, such as gentrification (Jones & Ponzini, 2018). As such, the emphasis on the economic value of culture has had a negative impact on the normative social capital of place‐based communities, exacerbating social inequalities (Steiner et al., 2015). Adverse effects seem rooted in the fact that investment is placed in short‐term, competitive, urban aesthetic interventions designed to attract external investment and visitors (Gilmore et al., 2019; Mathews, 2010). Alternatively, cultural policies that target the social value of culture foster mechanisms that contribute to increasing social and cultural capital, which potentially will improve wellbeing and alleviate social inequalities (Reidpath et al., 2005).

In an attempt to monetise social value, the UK Treasury defines social value as including ‘all significant costs and benefits that affect the welfare and wellbeing of the population, not just market effects’ (Treasury, 2022b). The political agenda to quantify and monetise the ancillary effects of embodied forms of cultural (e.g. active cultural practices) and social capital (e.g. networks) is at the centre of the argument to evidence and advocate for the instrumental value of culture.

### The social value of creative wellbeing

The instrumental value of culture and subsequent public investment is grounded in the ‘intermingled’ nature of capitals, in which cultural practices improve social and economic capitals and vice‐versa—for example, volunteering in a museum might lead to paid employment when volunteers use their learnt skills (cultural capital) and social networks (social capital) to get a job (economic capital), which will further develop social and economic capital (Fulkerson and Thompson, 2008). The interplay of three forms of capital (social, cultural and economic) is a valuable resource regarding social mobility attainment and preserving wellbeing and health (Bourdieu, 1982; Fancourt et al., 2018; Pinxten & Lievens, 2014). Hence, cultural value research seems to have developed under the assumption that cultural interventions are socially and economically valuable because they increase cultural, social and economic capital interchangeably (Beasley‐Murray, 2000).

The World Health Organization (WHO) Europe and the UK Department for Digital, Culture, Media and Sport (DCMS) published a review concluding the arts have an essential role in promoting health (Fancourt et al., 2020; Fancourt & Finn, 2019). Research examining relationships between social and cultural capital and creative health is still insufficient (Pinxten & Lievens, 2014), despite recent developments (Fancourt et al., 2018; Newman et al., 2013). Criticism of the review's source quality prompted calls for more rigorous and systematic reviews, aiming for reliable results (Clift et al., 2021). However, the creative health field is limited to the understanding of cultural value in diagnosed conditions (Ganga & Wilson, 2020), leaving unanswered the value of culture to improve wellbeing and reduce social and health inequalities. Our review aims to fill this critical gap in creative health research by focussing on creative wellbeing and healthy populations. While existing literature, including WHO Europe and the UK's DCMS, acknowledges the arts' role in health promotion, there's still a lack of understanding regarding cultural value in non‐diagnosed conditions. By focusing on healthy populations, our review seeks to elucidate how cultural interventions can enhance well‐being and address social inequalities. Through high‐quality impact evidence of creative well‐being, it aims to inform policy by identifying new strategies for practice (‘what works’) and raise new questions for further research in the scope of the sociology of health and wellbeing.

## METHODS

The review followed the Preferred Reporting Items for Systematic Reviews and Meta‐Analyses (PRISMA) statement (Page et al., 2021) and the Cochrane Handbook (Green et al., 2011) suggestion for reporting the characteristics of included studies—Studies, Data, Methods, Outcomes (Studies, data, methods and outcomes (SDMO)) approach (Munn et al., 2018).

The authors searched five electronic databases between 1st‐31st July 2022, including PubMed, MEDLINE, Web‐of‐Science, Cochrane and SCOPUS. The search strategy was designed, tested and refined in Web‐of‐Science. No text mining or automation tools were allowed and restrictions were as follows: (i) English; (ii) last 10 years; (iii) UK and Europe. Further relevant grey, online, or in‐press publications were identified through a manual search and a call for evidence led by the What Works Centre for Wellbeing (WWCW) (10th August to 26th August 2022).

A combination of the keywords place, art interventions and wellbeing was searched (Appendix 1). Our definition is grounded on Public Health England’s definition of communities as place‐based, where people share tangible and intangible heritage and lived experiences, as assets for social networking, community organisation, volunteering and developing skills and knowledge—‘building blocks for good health’ (Chatterjee et al., 2018). The Marmot review (2020) equally recommends, amongst other measures, healthy and sustainable places and communities to improve community capital and reduce social isolation.

Arts interventions were guided by creative health research, namely the Davies et al. (2012) definition of arts referenced within the WHO scoping review (Fancourt & Finn, 2019) and Davies and Clift (2022) arts and health glossary, which covers several means of active and receptive participation.^1^ Relevant to this review is the definition of arts interventions, such as attending museums and participating in community events. Art is valued beyond mere utility, emerging from an individual and collective creative process that fosters imaginative, aesthetic, emotional and intellectual responses for both producers and audiences (Davies & Clift, 2022).

Wellbeing is defined as a complex balance of multiple individual (e.g., physical and mental health, cultural capital, sense of purpose), social (e.g., social capital, civic engagement), economic (e.g., employment, housing, social welfare) and environmental (e.g., air quality, safety), factors that interact with each other and are dynamic over time. Wellbeing is both subjective (individual experiences) and objective (e.g., life expectancy, household income; Thomson et al., 2020). Bourdieu never described how to measure social capital (Pinxten & Lievens, 2014), which is problematic when aiming to understand the impact of arts interventions. Still, its institutionalised (formal and informal networks) and embodied (trust) forms can be captured. As such, wellbeing and social inequalities are analysed as proxies of social capital. This review uses the UK’s National Wellbeing Framework (ONS, 2022) domains and indicators to identify outcomes that relate to the social value of an art intervention. Primary and secondary qualitative, quantitative or mixed‐methods research from peer‐reviewed journal articles and grey literature sources were accepted. Eligibility was defined by the PICOS criteria, which were as follows: (i) Population—healthy humans of all genders with no age restrictions; (ii) Intervention—place‐based (city, town, borough, neighbourhood) interventions assessing the impact of arts and culture; (iii) Control—comparator time point or population; (iv) Outcome—at least one wellbeing or social inequalities outcome; (v) Study –design including at least one comparator (Appendix 2).

The authors independently conducted title, abstract and full‐text screening on the results from the databases and search engines and the call for evidence. Full eligible texts from both were screened based on inclusion criteria, then selected articles were screened for study designs that included a comparator component (pre‐post, intervention‐control, baseline and/or self‐reporting) as well as graded as either included/not included. Discrepancies were discussed and resolved by consensus (Appendix 3). Data extraction was conducted by one author and then checked by another, using a tool designed by WWCW. Quality assessment of quantitative research was done using a critical assessment framework taken from the *What Works Centre Guide to Evidence Review Methods* (Snape et al., 2019) and for qualitative research, a tool developed by the Critical Appraisal Skills Programme was used. Both these methods were chosen as they are robust, with clear guidance to aid the reviewer and minimise bias. Missing data was dealt with according to the Cochrane Handbook recommendation, by analysing only the available data (Green et al., 2011).

Data was then summarised in a structured narrative synthesis; this method was selected as statistical meta‐analysis and another form of synthesis, including meta‐ethnography for qualitative studies, was not feasible due to inconsistencies in research designs, outcomes measures and sample (Popay et al., 2006). The narrative synthesis aimed to determine the impact of the interventions and the factors shaping their implementation and success and was structured using the SDMO approach (Munn et al., 2018).

Study outcomes were analysed considering the 43 indicators that comprise the 10 Dimensions of Wellbeing (ONS). The mechanisms of change (success and drivers of inequalities) identified in the review fall under three subsections: (i) Processes—how participatory art and receptive practices might improve cultural access/participation and mitigate inequalities; (ii) People—how community participation led by local cultural leaders and experts increases social capital and how volunteering can foster active cultural participation/community engagement to generate positive cultural/social capital (Fulkerson and Thompson, 2008) and mitigate social/health inequalities (Farquhar et al., 2005); and (iii) Inputs—how the duration and resources invested in arts interventions lead to heterogeneous outcomes and how heritage is an asset used to engage participants in meaningful connections with place and individual narratives.

## FINDINGS

**FIGURE 1:**
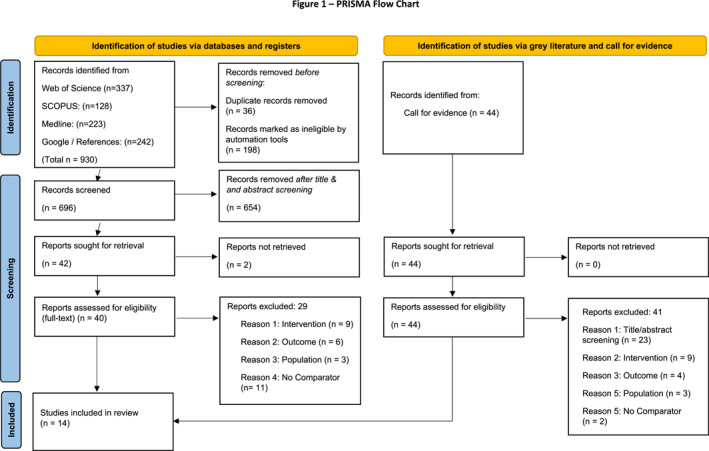
Preferred reporting items for systematic reviews and meta‐analyses flow chart. Page MJ, McKenzie JE, Bossuyt PM, Boutron I, Hoffmann TC, Mulrow CD, et al. (2021). The PRISMA 2020 statement: an updated guideline for reporting systematic reviews. BMJ 372, 71. 10.1136/bmj.n71.

The searches and call for evidence returned 974 results; after the screening, 14 studies remained for final inclusion. Search results and screening process are shown in the PRISMA flow chart (Figure 1) (Page et al., 2021).

The included studies spanned from 2013 to 2022 and were of interventions based in the UK (*n* = 10) and Europe (*n* = 4). They comprised quantitative (*n* = 6), qualitative (*n* = 2) and mixed methods (*n* = 6) designs, evidencing at least one wellbeing outcome (ONS, 2022). The included studies were categorised into three themes, each comprising two sub‐themes: *C* *ommunity*, heritage (*n* = 1) and music (*n* = 1); *Events*, music festival (*n* = 1) and Capital/City of Culture (*n* = 6); and *Museum*, volunteering (*n* = 2) and social prescribing (*n* = 3).

Most studies ranked moderate to high in quality. Two were found to be low quality as they reported insufficient detail on data collection and analysis. The first study, however, included an exploratory use of a novel technology for which thorough detail was provided (Echavarria et al., 2022). The second was a mixed‐methods study in which the qualitative methods were inadequately described, whereas the quantitative element was of moderate quality (Liu, 2014) (Appendix 4 and 5).

### Community

Study—included arts‐based research projects aimed to lower entry barriers and increase culture participation, amongst other wellbeing outcomes, through expertly facilitated engagement with local heritage. Data—a creative heritage‐driven intervention, sampling school children aged 9–12 (*n* = unspecified) in the Southeast of England (Echavarria et al., 2022); and a music project based at two youth clubs from disadvantaged backgrounds in the Northwest of England, which sampled (i) young people (*n* = 55) aged 13–18 (5% of group aged between 16 and (ii) young people (*n* = 23) aged 12–18 (30% of group aged between 16–18 Clennon & Boehm, 2014). Methods—Echavarria et al. (2022) used a pre‐post design to evaluate 10 workshops delivered by local artists, on children’s confidence, resilience and happiness. The measures used to capture these wellbeing constructs were not specified. Clennon & Boehm (2014) evaluated the impact of a year‐long music project delivered by experts and youth club volunteers by collecting qualitative data during and post‐intervention. Filmmaking as an art‐based method was used to capture emergent wellbeing outcomes. Outcomes—both studies demonstrated an improvement in subjective wellbeing outcomes, showing a positive impact on self‐esteem and confidence. Echavarria, et al. (2022) found increased happiness and resilience, while Clennon & Boehm (2014) found that emotional awareness and anger management strategies were both enhanced through active participation.

### Events

Study—a music festival, *La Notte Della Taranta* and six studies exploring the Capital/City of Culture, where interventions range from long‐term programmes (10 or more weeks) of participatory art practices and community‐led projects to one‐off large‐scale events and volunteering initiatives. These studies measured the impact of place‐based events on wellbeing outcomes. Data—Attanasi et al. (2013) surveyed attendees of the festival (*n* = 899,500) over five yearly editions. Culture, Place and Policy Institute (2018a) reported the impact of Hull’s year as the UK City of Culture. Steiner et al. (2015) provided a secondary analysis of the impact of the ECoC years in 14 countries. Neither of these provided a socio‐demographic description of their samples. Other studies on the impact of the ECoC sampled residents in Maribor, ECoC 2012, (*n* = 2,156, mean age = 49.22) and the wider population of Slovenia (*n* = 2,635, 53% female, mean age = 50.29) (Žilič Fišer & Kožuh, 2019); Liverpool, ECoC 2008, residents regarding *arts and culture participation* (*n* = 2252) (Liu, 2014) and *quality of life* (*n* = 592, 52% female, mean age = 44) (Liu, 2017); Riga, ECoC 2014, residents (*n* = 502), Latvia’s residents (*n* = 1045) and project organisers (*n* = 107). Methods—Attanasi et al. (2013) gathered data over 2 weeks of each *music festival* edition, enquiring about audiences’ *generalised trust* to measure the festival’s impact on immediate social capital. Of the six studies included in the Capital/City of Culture theme, three (Culture, 2018a; Liu, 2014; Žilič Fišer & Kožuh, 2019) implemented pre‐post designs, one used secondary retrospective data (Steiner et al., 2015) and two used primary post‐event data (Liu, 2017; Tjarve & Zemīte, 2016). Five studies used control groups, comparing host and non‐host cities (Steiner et al., 2015), neighbourhoods within a host city (Tjarve & Zemīte, 2016) and hosting city and country’s residents (Žilič Fišer & Kožuh, 2019). Cultural, Place and Policy Institute (Culture, 2018a, 2018b) assessed *happiness* and *life satisfaction* through non‐standardised measures, and Steiner et al. (2015) used the validated Life Satisfaction Approach. Five studies surveyed residents’ perceptions (Culture, 2018a; Liu, 2014, 2017; Tjarve & Zemīte, 2016; Žilič Fišer & Kožuh, 2019). Liu (2014) used 21 non‐standardised measures informed by the mega‐events literature. One study used semi‐structured interviews (Tjarve & Zemīte, 2016), and another used community‐based workshops (Liu, 2017).

Most/City of Culture studies reported improvements in subjective wellbeing outcomes, including *life satisfaction*, *feeling worthwhile*, *happiness* and *mental wellbeing*. However, in some instances, these improvements were short‐term (Culture, 2018b) or lacked long‐term follow‐up evidence (Tjarve & Zemīte, 2016). Some studies also reported improvements in *what we do* (volunteering, arts and culture participation) (Culture, 2018b; Liu, 2014; Tjarve & Zemīte, 2016) *where we live* (belonging to neighbourhoods) (Žilič Fišer & Kožuh, 2019) and *education and skills* (human capital) (Culture, 2018b). Steiner, et al. (2015) demonstrated lower *life satisfaction* in host versus non‐host cities. Liu (2014) found an instantaneous adverse impact on *where we live* (belonging in the neighbourhood); however, longitudinal analysis showed an improvement (Liu, 2017). Liverpool ECoC 2008 studies (Liu, 2014, 2017) demonstrated adverse outcomes on *our relationships* (loneliness), *what we do* (unemployment, dissatisfaction with leisure time), *where we live* (crime, belonging to neighbourhood), and *economy* (disposable income, public sector debt). City/Capital sub‐theme studies’ adverse outcomes seem to be impelled by the following drivers of inequalities (Abdallah et al., 2017): insufficient community‐based cultural activities, anti‐social behaviour, unemployment and excessive tourism (Liu, 2014, 2017; Steiner et al., 2015).

### Museum

Included Studies observed how museums are anchor institutions fostering wellbeing and mitigating social inequalities through volunteering (*n* = 2) and social prescribing (*n* = 3). Data—Thomson, Elsden and Chatterjee (Thomson et al., 2020) sampled young adults aged 18–25 years, older adults aged 50 plus, and people experiencing *health* challenges, *loneliness*, or long‐term *unemployment* (*n* = 40), utilising a series of training sessions, based in London. Warby, Garcia and Winn (Warby et al., 2013) assessed 10 weeks of museum‐based training followed by a 6‐week placement, based in Manchester. The sample included young people aged 18–25, people aged 50+, armed forces veterans and adults with long‐term *unemployment*, all of whom had levels of wellbeing below the national average (*n* = 231). Thomson et al. (2018) assessed a 10‐week‐long intervention across seven museums in London and Kent. The sample included adults aged 65–94 referred by healthcare organisations due to risk of *loneliness* (*n* = 115, 63% female, 82% White British). Todd et al. (2017) recruited participants from Thomson et al. (2018) sample (*n* = 20, 50% female). Dodd and Jones (2014) reported on three different projects, sampling (i) older adults (*n* = 93), (ii) school‐aged students (*n* = 5) and (iii) young people aged 9–24 years (*n* = 113). All three projects were a single session based in a museum or art gallery in the East Midlands. Methods—four museum‐based studies provided evidence from pre‐post designs (Dodd and Jones, 2014; Thomson et al., 2018, 2020; Warby et al., 2013). Todd et al. (2017) qualitatively explored the impact of Thomson et al. (2018) post‐intervention data. In one of the projects reported by Dodd and Jones (2014), findings were compared to a national pilot study of the Museum Wellbeing Measures Toolkit. Validated scales within this theme included the Warwick‐Edinburgh Mental Wellbeing Scale (Thomson et al., 2020; Warby et al., 2013), the UCL Museum Wellbeing Measure (Thomson et al., 2020), the Museum Wellbeing Measure for Older Adults (Thomson et al., 2018) and Positive and Negative Wellbeing Umbrellas (Dodd and Jones, 2014). Four studies collected qualitative data using semi‐structured interviews (Clennon and Boehm, 2014; Thomson et al., 2018, 2020; Tjarve & Zemīte, 2016; Todd et al., 2017; Warby et al., 2013). Dodd & Jones (2014) use response cards and questionnaires to evidence knowledge and awareness. Some studies included reflective methods such as diaries or maps (Thomson et al., 2018, 2020; Todd et al., 2017). Outcomes—the two volunteering‐themed studies demonstrated improvements in *subjective wellbeing* (feeling worthwhile, mental wellbeing), *our relationships* (loneliness, people to rely on), *what we do* (volunteering^2^) and *education and skills* (human capital). However, Todd et al. (2017) reported a decline in *mental well‐being—*qualitative data suggested this could be due to an increase in tiredness from the sessions becoming more intellectually demanding. Warby et al. (2013) showed improvements in *life satisfaction* (subjective wellbeing) and *arts and culture participation* (what we do). Studies in the social prescribing sub‐theme showed improvements in *subjective wellbeing* (mental wellbeing, happiness), with one qualitatively demonstrating improvement in self‐esteem and confidence as well as a positive impact on *health* (mental, physical) and *our relationships* (loneliness), though not all participants wanted to make social connections (Todd et al., 2017). One of the projects reported by Dodd & Jones (2014) showed improvement in *education and skills¸* through enhancing knowledge and awareness.

## DISCUSSION

This review aimed to answer the research question, ’What is the social value of place‐based arts and culture interventions at an individual and community level in the UK and Europe?’ The narrative synthesis of the 14 included studies provides a nuanced understanding of the value of culture in improving wellbeing outcomes and reducing social inequalities (Munn et al., 2018). It mapped the wellbeing outcomes of the included studies against 20 indicators of the UK’s National Wellbeing Framework (ONS, 2022) (Tables S1 and S2) and analysed the mechanisms of change (success and drivers of inequalities) and resources that underpin creative wellbeing. The key findings are discussed according to the situated significance of place, participatory processes and mechanisms of relative success.

### Place & asset‐based approaches

Five studies discuss museums as anchor institutions, where participants are aesthetically and intellectually stimulated by the collections and experience social networking with peers and museum staff (Dodd and Jones, 2014; Thomson et al., 2018, 2020; Todd et al., 2017; Warby et al., 2013). The combination of relational participatory practices, museum spaces and resources generates opportunities for contemplative experiences, positive social interactions and the development of new knowledge and skills, leading to an increase in wellbeing outcomes and a decrease in social inequalities (Chatterjee and Noble, 2016; Mangione, 2018; Thomson et al., 2018). The five studies evidence how museums contributed to reducing social isolation in disadvantaged communities and act as preventive and restorative actions in the social determinants of health (Dodd and Jones, 2014; Thomson et al., 2018; Todd et al., 2017; Warby et al., 2013).

Six studies on the events theme have much more nuanced impacts. According to Klijs et al. (2017), social relations act as a buffer for the adverse effects of neighbourhood deprivation on psychologically‐related quality of life. Events that are culturally bounded and hyper‐local (within the neighbourhood) enable communities to celebrate their shared culture and address challenges through participatory creative processes. Culturally rooted place‐based events lead to an increase in trust among participants, generate social capital and increase individual and community wellbeing, particularly within historically disengaged communities (Attanasi et al., 2013; Culture, 2018a; Liu, 2017; Tjarve & Zemīte, 2016; Žilič Fišer & Kožuh, 2019). Four studies, however, provide evidence on how the City/Capital of Culture might exacerbate social inequalities, as large‐scale, city centre‐focussed events and physical infrastructure developments benefit the most privileged communities, exacerbating social inequalities and life satisfaction disparities across the city (Steiner et al., 2015).

Intangible heritage assets are used across nine studies, engaging participants with place, articulating individual narratives and developing skills (Attanasi et al., 2013; Clennon and Boehm, 2014; Culture, 2018a; Dodd and Jones, 2014; Echavarria et al., 2022; Thomson et al., 2018, 2020; Todd et al., 2017; Warby et al., 2013). For example, in Hull UK CoC 2017, half of the commissioned programme was inspired by the city’s heritage. Nearly all (91.3%) of participating audiences felt that using an arts‐based approach helped to break down barriers and fostered appreciation, understanding and increased knowledge of the city’s heritage and history (Culture, 2018b).

Across the three themes, effective networking, partnership and cross‐sector collaboration with existing public assets expertly facilitated were integral in ensuring sustainability and long‐term legacies. The *Young Musicians for Heritage Project* (Clennon and Boehm, 2014) used heritage‐based creative activities led by ‘social capital builders’ (Adams, 2020), aligned with the group's needs and interests, creating a new outlet for participants, some of whom faced challenging emotional/social issues, to develop skills (e.g. networking, leadership, creativity) and improve their subjective wellbeing. Young people worked with anchor institutions such as Crewe City Hall and Crewe Heritage Centre, reconnecting them with their local heritage and increasing their interest and participation in arts and culture.

Echavarria et al. (2022) creatively engaged children to produce place‐based narratives of their daily journeys between home and school, which became Augmented Reality (AR) Maps that the children and their families could experience and share. This study combines arts‐based and novel technologies to empower children to interpret, engage and communicate their viewpoints about cultural heritage. The reinterpretation of place‐based narratives facilitates connections between people, objects, sites and events in the urban landscape (Echavarria et al., 2022), while improving individual and community wellbeing. It is categorised within the *Community* theme and communicates the value of anchor institutions such as schools and museums. At the end of the project, a ‘celebration’ took place at *Hove Museum*, where all children, their families and friends accessed the AR Map for the first time. Results show a 45% increase in children feeling very happy, an 18% increase in children reporting liking themselves, a 15% increase in feeling liked by other people and a 15% increase in children reporting that they coped with difficult situations happily or very happily.

### Cultural capital: Active & receptive participation as mechanisms of success

Participation is a concept that encompasses multiple modalities of engagement (Huybrechts, 2014). Active participation in community settings through participatory arts practice emerges in the included studies as a common mechanism of success to improve multiple wellbeing outcomes (Grundy & Boudreau, 2008). These art practices assume multiple forms across the different studies, ranging from co‐design and co‐production, storytelling, volunteering training, cultural heritage research and interpretation, having in common the participants as producers (Bishop, 2012).

Included studies provided evidence of the relational and creative processes that lead to positive wellbeing outcomes, namely learning new skills, building relationships, enhancing the sense of community and belonging, developing social capital, creating and sharing narratives, co‐production and exchange of ideas (Klijs et al., 2017; Science, 2008; Thomson et al., 2018). The included studies provide evidence on the effects of participatory art practices as community‐ and participant‐centred processes, flexible, encourage strong partnership working and are thus valuable for engaging individuals from disadvantaged backgrounds.

Clennon & Boehm (2014) argue that participatory art practices have more long‐lasting eudemonic effects than hedonic wellbeing. Participatory art practices not only enable enjoyment but are a vehicle to make sense of and symbolically express difficult feelings and challenging life experiences by reinterpreting those artistically as a process of self‐empowerment (Clennon and Boehm, 2014; Dodd and Jones, 2014; Thomson et al., 2018; Todd et al., 2017). Storytelling and narratives, as fundamental ways to make sense of reality, have been recognised as engagement strategies with the surrounding world (e.g. collections, heritage sites), but also to meaningfully articulate life experiences and forge connections with others within the community and foster social capital (Clennon and Boehm, 2014; Culture, 2018a; Echavarria et al., 2022). *Let's Create* (England, 2020) underscores the need for more research into the impact of non‐asset‐based interventions on creative wellbeing. Further research is needed on the importance of community‐based interventions, including inclusive and diverse cultural activities accessible to all. A community‐based approach will provide a broader perspective on how arts and culture are defined and valued in different contexts.

The Culture, Place and Policy Institute (2018b) provided evidence of the adverse effect of social and health inequalities on cultural access and participation, particularly regarding disability, ethnicity, age and social deprivation (McLennan et al., 2019). Only slightly over 25% of the Hull residents were ‘limited a lot’ by health conditions or disability, 40% of Black, Asian and minority ethnic and less than 35% of younger people (24 and under) felt their lives and communities were represented in the cultural programme. These perceptions of lack of representation were mirrored in lower levels of cultural participation when compared with other population segments. Equally, 45.1% of Hull’s population residing in the 10% most deprived areas struggled to/did not access cultural activities. The same findings are reported by Liu (2017) regarding the Liverpool ECoC 2008. Despite the residents’ support of the event, those from the 10% most deprived areas (Kirkdale and Knotty Ash) did not feel represented. The location of residence in the city had a significant statistical influence on residents’ perceptions of the impact of ECoC. Communities on the ‘geographical peripheral’—that is, furthest away from the city centre with limited public transport—and those defined as socially deprived—have lower cultural, social and economic capital (Bourdieu, 2018a), had lower cultural participation figures (Aigburth—78%; City Centre—72%; Knotty Ash—59%; Kirkdale—56%) and, as such, experienced lower positive cultural impacts of ECoC. Both had experienced lower levels of investment in terms of physical renewal/urban regeneration and cultural programming. Qualitative data points to dissatisfaction with the perceived lack of dedicated cultural programmes for children and young people and in addition, cost, timing, location and lack of information were highlighted as barriers to cultural participation (Liu, 2014). Overall, the ECoC did not contribute to tackling the impact of inequalities in cultural access and participation with mixed value to wellbeing (Attanasi et al., 2013; Culture, 2018b; Liu, 2014, 2017; Steiner et al., 2015; Tjarve & Zemīte, 2016; Žilič Fišer & Kožuh, 2019).

On the other hand, intensive engagement activities, such as volunteering and museums’ volunteer training, stimulated participants to visit museums and galleries and pursue further learning opportunities, increasing their cultural capital (Chatterjee, 2016; Warby et al., 2013).

### Social capital: Networking & volunteering as mechanisms of success

Social capital theory and research emerge in some of the included studies without an explicit definition, which might indicate the normalisation of the scientific concept (Kuhn, 1962). Despite its contested meaning, we discussed individuals, resources, relationships and networks and how place‐based arts interventions foster social capital both as a resource (Bourdieu, 2018a) and as a norm (Fulkerson and Thompson, 2008; Treasury, 2021, 2022a).

Across the studies included in the themes *Community* and *Museums*, a common mechanism of success was the value of cognitively stimulating social interactions. Community or museum‐based participatory art practices are, above all, social interactions expertly curated by facilitators (e.g., artists and museum experts) throughout a period of time. The dynamic nature of those social interactions might foster or hinder wellbeing and social outcomes (Todd et al., 2017). The investment in engaging and empathic professional relationships (Camic et al., 2017) and time to allow social ties (Granovetter, 1973) to be developed leads to tailored interventions that are co‐designed, owned and shared with participants (Clennon and Boehm, 2014; Thomson et al., 2018, 2020; Warby et al., 2013), fostering social and cultural capital.

Studies from the Museums theme evidence that interactions with museum staff, artists, educators and volunteers are intellectually stimulating and pleasant experiences for participants, developing resource social capital (Dodd and Jones, 2014; Thomson et al., 2018, 2020; Todd et al., 2017; Warby et al., 2013). Participants felt intellectually challenged and emotionally engaged by the experts and aesthetically delighted by the museums and their collections (Chatterjee, 2016; Thomson et al., 2018). However, representation and inclusiveness of traditionally less engaged communities are ongoing museum challenges. Training of museum volunteers, using strategies such as peer‐support and mentoring, is used as an approach to close the gap between disadvantaged and vulnerable communities and more elitist museum expertise (Thomson et al., 2018). Time and resources need to be embedded from the onset to allow the development of well‐integrated and strong social ties in the community (Granovetter, 1973).

Clennon & Boehm’s (2014) study (included in the *Community* theme) corroborates the role of community participatory art practices where peer mentoring and leadership skills training are also incorporated to tackle social inequalities. These strategies supported young participants’ independence, illustrated by the emergence of youth leadership groups. The opportunities to participate in cultural networks with experts, community leaders and peers generate normative and resource social capital.

Five studies included in the review, namely Thomson et al. (2020, 2018), Dodd & Jones (2014), Warby et al. (2013), Culture, Place and Policy Institute (2018b), Liu (2014, 2017) and Clennon & Boehm (2014) provide evidence on how volunteering might revert historical processes of inequality through the development of normative and resource social capital—volunteering in a museum can lead to paid employment when volunteers use their learnt skills and social networks to get a job (Fulkerson and Thompson, 2008). Employment is one of the most significant determinants of health, as it enables financial security, social mobility, social networks and personal growth (Marmot et al., 2008). Furthermore, volunteering demonstrates that the value of creative wellbeing is grounded in the interchangeable nature of capitals (Bourdieu, 1983, 2018b).

Cultural mega‐events increase residents' uptake of volunteering (Liu, 2017). However, further granular research (or better reporting) would be needed to understand the social capital generated by volunteering in cultural mega‐events. Museums effectively provide volunteering opportunities (Dodd and Jones, 2014; Thomson et al., 2018, 2020). Building social networks and doing something purposeful with the available time is perceived as restorative (Deery et al., 2011; Edwards, 2005), socially active and intellectually stimulating with long‐term sustained wellbeing improvement. At the same time, volunteering develops and restores social relationships in the community, helping to reduce social isolation and generating normative social capital (Clennon and Boehm, 2014). Again, further research would be needed on how volunteering in the community generates social value that would mitigate social inequalities, especially within disadvantaged communities.

### Economic capital: Resources as mechanisms of success

The heterogeneity of investment in place‐based arts interventions, considering duration (short‐to long‐term), funding and assets (e.g. cultural organisations, collections, cultural heritage), leads to heterogenic outcomes. Long‐term interventions with target recruitment are common mechanisms of success in all studies as a resource to facilitate wellbeing outcomes and social inclusion. The included interventions ranged from 1 to 3 years, although there was a lack of detail on the length of the hyper‐local interventions of mega‐events. Recommendations across the three themes address the need for long‐term intervention to sustain wellbeing outcomes over time.

The studies included in the *Events* theme reported both short and long‐term interventions, stating that the economic resources were proportionally inverted to the length of the intervention. The *Road Map* programme, Riga ECoC 2014, for example, involved ‘a large number of small initiatives with limited funding’, that ‘might be among one of the most sustainable results with significant influence on the local development of the city’ (Tjarve & Zemīte, 2016). This issue is particularly problematic in the City/Capital of Culture, which requires ‘significant’ public (on average 77.5%) and private investment (on average 13%). Short‐term large‐scale events (e.g., final concert) tend to absorb most of the budget (roughly 70%) (Attanasi et al., 2013). While Qualitative Comparison Analysis led by Tjarve and Zemīte (2016) demonstrated the value of hyper‐local cultural organisations and cultural heritage, as drivers of community cultural life. In this case, the economic capital does not seem to increase creative wellbeing's social and cultural value.

In the Museums' subtheme, Warby et al. (2013) provided data on the funding invested in the intervention and its social return. *IF* (Warby et al., 2013), a museum‐based volunteering programme generated a social and economic value of approximately £2 million and approximately £3.50 of social and economic return was created for each £1 invested. On the social prescribing subtheme, Thomson et al. (2018) and Todd et al. (2017) argue that museum‐based social prescribing is ‘low cost’ and ‘cost‐effective’, but no specific figures are disclosed.

Attanasi et al. (2013) argued that the investment, financial and otherwise, in the festival's organisation is a valuable return on investment as the festival leads to the socioeconomic development of the host villages. This is due to the visitors' economy and the strengthening of cultural identity, as the event is grounded in local cultural intangible heritage. The study argues for the correlation between local tradition being celebrated by residents and tourists and the growth of social capital, innovation, cultural tourism, economic development and local identity revitalisation. However, the study is focused on trust and instantaneous social capital, failing to provide evidence of the festival’s effectiveness in preserving and fostering intangible cultural heritage (Attanasi et al., 2013).

However, across all studies, there is no indication of scaling‐up of programmes and interventions. The majority of studies provided poor descriptions of the interventions themselves, particularly regarding inputted resources, which poses a challenge in terms of replicability and scalability (Clift et al., 2021).

### Limitations

Our review acknowledges the enduring challenge of unsatisfactory evaluation methodologies in the field, compounded by its increasingly multidisciplinary nature (Belfiore, 2006; Clift et al., 2021). We highlight that most studies focus on short‐term individual and community wellbeing outcomes due to the resource‐intensive nature of long‐term studies, which also raises attribution issues. The heterogeneity in methodological approaches within this multidisciplinary field confounds evidence synthesis, especially considering the traditional hierarchies of evidence that privilege randomised controlled trials. Our review advocates for a balanced methodological approach that combines methodological robustness with qualitative and arts‐based designs to effectively capture the social determinants of health.

## CONCLUSION

The results of this review illustrate the impacts of arts interventions on wellbeing and social inequalities in the UK and Europe. Findings suggest that socially cohesive communities (social capital) and active cultural practice across the life course (cultural capital) are social and cognitive protective factors that enable health and wellbeing.

The review introduced a distinction between two typologies of art interventions (i) short‐term, large‐scale, aesthetically accessible, free activities, staged in the city centre (e.g., concerts); and (ii) hyper‐local, co‐created, intellectually challenging, expertly facilitated, longer‐term, and heritage‐focussed. Findings suggest that the former can foster receptive participation due to increased cultural offers and infrastructures, shared celebratory feelings of trust and improved image and identity, but without lasting effects. Alternatively, the latter seems to increase subjective wellbeing, active participation and volunteering through place‐based narratives, co‐creation and other participatory art practices, promoting social cohesion and community networks and improving skills. Adverse and neutral outcomes are also evidenced, namely the decline in community cohesion and neutral impact on life satisfaction and happiness in the UK and Europe.

The museum and the community are favourable contexts for hyper‐local, engaging, medium‐to‐long term art and cultural practices. The museum is a rich heritage setting that can be a safe and stable environment for social prescribing programmes, with the infrastructure to foster cross‐sector collaborations with health and social care services and community cultural leaders. Museum‐based volunteering effectively developed cultural and social capital, with longer‐term health, wellbeing and employability improvements. Interventions with community expertise and leadership were able to create heritage‐informed, intellectually challenging and co‐created activities with children and young people with historically low levels of participation in arts and culture. Investment in cultural infrastructure, urban regeneration and extensive cultural mega‐event programmes fosters passive participation, leading to immediate, short‐lived positive outcomes that can exacerbate social inequalities in the long term. Despite evidence of the contribution of place‐based arts interventions in alleviating the social determinants of health, caution is needed when considering their impact on the underlying causes of structural social inequalities, given the lack of controlled studies.

The review’s contribution is twofold, through (i) providing evidence of the effectiveness of community‐based participatory art practices to improve wellbeing and tackle the social determinants of health and (ii) providing an insight into the mechanisms that are more efficient in achieving those outcomes and improving understanding of the drivers of inequality. Current conclusions are insightful but limited. Supplementary, high‐quality mixed‐methods research is needed to demonstrate long‐term impacts and produce scalable road maps of what works for wellbeing. Particular attention is needed to the heuristic quality of participatory art practices, both in the delivery and research of the intervention, as distinct from other forms of social engagement. A strength of the present review is its contribution to the knowledge of specific individual and community wellbeing outcomes resulting from participating in arts, culture and heritage activities, particularly the impact of community projects within cultural mega‐events and methodologically robust museum‐based programmes. Through wider knowledge exchange, flexible and useable creative intervention principles may be applied in multiple community settings.

## AUTHOR CONTRIBUTIONS

**Rafaela Neiva Ganga**: Conceptualisation (lead); data curation (supporting); formal analysis (supporting); funding acquisition (lead); investigation (lead); methodology (lead); project administration (lead); supervision (lead); validation (lead); visualisation (supporting); writing – original draft (lead). **Laura Davies**: Data curation (lead); formal analysis (equal); investigation (supporting); validation (supporting); visualisation (lead); writing – original draft (supporting). **Kerry Wilson**: Funding acquisition (supporting); writing – original draft (supporting). **Margherita Musella**: Conceptualisation (lead); formal analysis (supporting); investigation (supporting); writing – review & editing (equal).

## CONFLICT OF INTEREST STATEMENT

The authors declare that they have no competing interests.

## ETHICS STATEMENT

Ethics approval and consent to participate.

## Supporting information

Supporting Information S1

Supporting Information S2

Supporting Information S3

Supporting Information S4

Supporting Information S5

Table S1

Table S2

## Data Availability

The datasets used and/or analysed during the current study are available from the corresponding author upon reasonable request.

## ENDNOTES

^1^
Active participation refers to creating art (e.g. producing music). Receptive participation refers to experiencing art (e.g. attending a concert as part of an audience) (Clift et al., 2021).

^2^

*Arts and culture participation* and *volunteering* are considered both mechanisms of success and well‐being outcomes per the UK’s National Well‐being Framework (ONS, 2022).
