# Supplementary material for: The social value of place‐based creative wellbeing: A rapid review and evidence synthesis
Source: Sociol Health Illn. 2024 Aug 17;47(1):e13827. doi: 10.1111/1467-9566.13827 (PMC11684509; doi:10.1111/1467-9566.13827)
Supplement: Supplementary file 1 — Supporting Information S1 [file SHIL-47-0-s002.docx]

The following sensitive search terms were used as keywords:

*((((((((((CU=(UK OR ‘United Kingdom’ OR England OR Scotland OR Wales OR ‘Northern Ireland’ OR Europe OR Austria OR Belgium OR Czechia OR ‘Czech Republic’ OR Denmark OR Estonia OR Finland OR France OR Germany OR Greece OR Hungary OR Iceland OR Italy OR Latvia OR Liechtenstein OR Lithuania OR Luxembourg OR Malta OR Netherlands OR Norway OR Poland OR Portugal OR Slovakia OR Slovenia OR Spain OR Sweden OR Switzerland)) AND ALL=(place-bas* OR ‘place bas*’ OR place OR local OR capital OR cit* OR town* OR neighbo*rhood* OR borough* )) AND ALL=(art OR cultur* OR ‘art* intervention*’ OR ‘cultur* intervention*’ OR ‘art* program*’ OR ‘cultur* program*’ OR participation OR ‘cultur* participation’ OR ‘co-production’ OR ‘participatory art’ OR volunteering OR engagement OR co-creat* )) AND ALL=(event OR ‘mega-event’ OR ‘mega event’ OR ‘large-scale event’ OR ‘large scale event’ OR ‘major event’ OR major-event )) AND ALL=(‘social value’ OR wellbeing OR well-being OR wellness OR ‘psychological health’ OR ‘community cohesion’ OR ‘social *clusion’ OR satisf* OR dissatisf* OR belonging OR worthwhile OR happ* OR anxi* OR ‘quality of life’ OR ‘mental health’ OR inequalit* OR disparit* OR loneliness OR ‘job* satisfaction’ OR ‘relationship satisfaction’ OR autonomy OR resilience OR ‘self control’ OR self-control OR ‘self concept’ OR self-concept OR ‘self respect’ OR self-respect or ‘self esteem’ OR self-esteem OR ‘self attitude’ OR self-attitude OR ‘sense of coherence’)) NOT ALL=(relig* OR pray OR worship OR doctrine OR theology )) NOT ALL=(sport* OR ‘physical activity’ OR exercise OR diet OR nutrition* OR eating or diet )) NOT ALL=(parks OR greenspace* OR ‘green space*’ OR bluespace* OR ‘blue space*’ OR nature OR ‘natural world’ OR ‘natural environment’ OR countryside OR climate OR marine OR ‘environmental education’ )) NOT ALL=(tour* OR trav* )) NOT ALL=(market* or econom* OR retail OR finance )) NOT ALL=(‘health settings’ OR hospital OR clinic OR ‘health centre’ )*
