# Supplementary material for: The social value of place‐based creative wellbeing: A rapid review and evidence synthesis
Source: Sociol Health Illn. 2024 Aug 17;47(1):e13827. doi: 10.1111/1467-9566.13827 (PMC11684509; doi:10.1111/1467-9566.13827)
Supplement: Supplementary file 2 — Supporting Information S2 [file SHIL-47-0-s003.docx]

## Appendice 3 - Population Intervention Control Outcome (PICO) criteria

| Population/Setting | Any |
| --- | --- |
| Intervention | place-based arts and culture interventions |
| Comparison | N/A |
| Outcome | At least one wellbeing or social inequalities outcome |
| Study types included in the screenings | No restrictions |
| To be excluded | - Non-arts or cultural interventions - Non-place-based / Non-cultural event - Languages other than English |
