# Supplementary material for: The social value of place‐based creative wellbeing: A rapid review and evidence synthesis
Source: Sociol Health Illn. 2024 Aug 17;47(1):e13827. doi: 10.1111/1467-9566.13827 (PMC11684509; doi:10.1111/1467-9566.13827)
Supplement: Supplementary file 3 — Supporting Information S3 [file SHIL-47-0-s007.docx]

## Appendix 4 – Inclusion criteria

| **Parameter** | **Inclusion criteria** |
| --- | --- |
| Condition | Healthy population |
| Publication dates | 2002-2022 |
| Publication types | No limit, including policy reports and grey literature |
| Study types/designs | *Primary and secondary research:* Evaluations, RCTs/clinical trials, observational studies (cohort (before and after) and case-control studies), pragmatic trials, impact assessment, meta-analysis |
| Study subjects/participants | Humans |
| Age | No limit |
| Gender | Male, female, non-binary |
| Interventions | Assess the impact of arts and culture interventions /events/mega events in individuals (wellbeing) and in society (social inequalities) |
| Outcome(s) | Improve wellbeing outcomes and social inequalities |
| Follow-up time | NA |
| Language | English |
| Research disciplines | Humanities and Social Science |
| Geographic location/country of study | UK and Europe |
| Settings/context | City, town, neighbourhood, borough |
